# Design and characterization of polymeric microneedles containing extracts of Brazilian green propolis

**DOI:** 10.3762/bjnano.13.42

**Published:** 2022-06-08

**Authors:** Camila Felix Vecchi, Rafaela Said dos Santos, Jéssica Bassi da Silva, Marcos Luciano Bruschi

**Affiliations:** 1 Laboratory of Research and Development of Drug Delivery Systems, Postgraduate Program in Pharmaceutical Sciences, Department of Pharmacy, State University of Maringa, Maringa, Brazil

**Keywords:** development, mechanical, microneedles, propolis extract, technology

## Abstract

Microneedles (MNs) are a means to break the protective skin barrier in a minimally invasive way. By creating temporary micropores, they make biologically active agents available in the skin layers. Propolis (PRP) is a gum resin with a complex chemical composition, produced by bees *Apis mellifera* L. and showing several therapeutic properties (i.e., antibacterial, antiviral, antifungal, anti-inflammatory, healing, and immunomodulatory properties). The administration of PRP extracts by conventional routes has some disadvantages, such as running off over the skin in liquid or emulsion form. When taken orally, the extracts have a strong and unpleasant taste. The aim of this work was to fabricate and characterize microneedles containing polyvinyl alcohol, polyvinylpyrrolidone, poloxamer P407, and an ethanolic or glycolic extract of PRP. Also, the obtained structures were microscopically and mechanically characterized. The results of the mechanical analysis showed that formulations containing 3% of P407 presented the highest compression values in a hard surface, which was also confirmed by the height and base values of the morphological analysis and by the microscopy images. It was possible to design MNs and select the best formulations for future tests. MNs containing an ethanolic extract of PRP showed to be better structured than MNs containing a glycolic extract of PRP. The MNs obtained in these studies proved to be a promising platform for the topical application of PRP.

## Introduction

In recent decades, microneedle devices have been widely used for non-invasive dermal delivery of various drugs [1–3]. Microneedles (MNs) are large enough to penetrate and open small holes only in the stratum corneum and the viable epidermis, without reaching the nerve endings that are in the dermis [4–5]. The perforation of the stratum corneum enables the release of bioactive molecules in the epidermis, which then reach the dermis and blood capillaries by diffusion [6]. This entire process occurs in a non-invasive, painless, and bleeding-free way [7]. MNs can be organized into single structures or arranged in small arrays to mediate the localized release of therapeutic molecules [8]. Regarding the design, MNs can be categorized into solid, hollow, dissolvable, and coated MNs, according to the differences in the permeation mechanism [6,9–10].

Nanocarriers can be used together with polymeric MNs in a synergistic therapy. The nanocarriers can immediately come into contact with the stratum corneum with the help of polymeric MNs, enhancing the transdermal drug delivery of the drugs. Furthermore, these polymeric MNs can encapsulate several types of nanocarriers, making it a unique system with different activities [11]. Solid MNs are used for pre-treatment of the skin. They serve only to create micropores, increasing permeability and facilitating the administration of the drug. The drug will be inserted over the holes created and will be taken, by diffusion, to the innermost layers to have its systemic action [5,12]. Coated microneedles are solid MNs made of inert material and coated with a formulation containing the drug to be administered [5,13]. After skin perforation, this lining is retained in the epidermis and, after a period, the MN system is removed [6].

Dissolvable MNs are composed of a biodegradable matrix containing the bioactive agent. When they come in contact with aqueous fluids in the skin, these needles dissolve, releasing the drug and degrading. As they are dissolvable, they do not need to be removed and do not generate residues, eliminating the risk of infections and local irritations when the microneedles are broken or incompletely removed. Hollow MNs have a reservoir inside, which contains the bioactive agent. They are used to penetrate the skin, inject their charge through the epidermis, and are later removed [6,14].

The application of MNs creates a transport pathway for the delivery of molecules, crossing external barriers that limit the introduction of molecules into the target tissue. Furthermore, MNs are very versatile and are considered less painful, less harmful, and more safe than conventional needles [5]. In general, MNs cause less damage than other larger, more invasive devices, such as hypodermic needles [15]. The creation of micropores is a physical technique that can be used to increase transdermal drug delivery by creating micropores in the stratum corneum before or during application, which can increase the permeation of certain molecules by up to 200 times [16]. In addition to being a minimally invasive route, transdermal drug delivery has low drug absorption variability among patients, since the cutaneous metabolism is significantly lower than the gastrointestinal one. Despite the great therapeutic potential, the use of this pathway is limited by the low permeation of molecules through the stratum corneum, the outermost layer of the skin, which works as a barrier, blocking the transport of drugs through the subcutaneous tissue. To overcome this difficulty, microneedles have been developed to cross the stratum corneum and enable the use of the transdermal route in different therapies [6].

Propolis (PRP) has already been studied in wound healing when incorporated in many vehicles, such as ointments, emulsions, hydrogels, films, or as hydroalcoholic or glycolic extracts. PRP exhibits important pharmacological activity, already proven in several studies, in addition to being a biologically safe compound [17–21]. This natural drug is also widely used as antimicrobial agent, immune system strengthener, and anticancer drug in the form of its ethanolic or glycolic extracts. It is a strongly adhesive gum resin, which is collected, processed, and used by honey bees (*Apis mellifera* L.) [17,22–23].

PRP extracts have been reported to enhance some antibiotic effects, attributing the antibacterial activity of propolis mainly to flavonoids or synergisms among some components [24]. The ethanol extract of PRP has several pharmacological activities, such as antiviral, antibacterial, antifungal, anti-inflammatory, anesthetic, cytostatic, hypotensive, and immunostimulatory properties [25]. Glycolic extracts can be an alternative in situations where the use of alcoholic extracts is inappropriate. Propylene glycol can be used as solvent, extractant, and preservative in a variety of pharmaceutical, cosmetic, and food formulations. It is compatible with several cosmetic and pharmaceutical bases, in addition to having an antiseptic capacity similar to that of ethanol and lower toxicity [26–27].

In our previous studies, polymeric systems composed of polyvinyl alcohol (PVA), polyvinylpyrrolidone (PVP), and poloxamer 407 (P407) were obtained and characterized. P407 could improve structuring and rapid dispersion of polymeric matrices, which showed promising physicochemical characteristics for potential application as nanostructured platforms for controlled drug delivery [28–30]. To our knowledge, MNs containing PRP have not been proposed before. Therefore, the aim of this study was to design microneedles composed of PVA, PVP, and P407 for the delivery of ethanolic or glycolic extracts of Brazilian green PRP. A 3^2^ full-factorial design was utilized to determine the influence of P407 and PRP extract on the morphology and mechanical characteristics of MNs. They were characterized macroscopically, microscopically, and regarding size and texture, yielding an improved MN formulation for each type of PRP extract.

## Results and Discussion

### PRP quality assessment

The PRP sample was obtained from an apiary in the northwest region of Paraná. It had a characteristic aromatic odor, resin gum appearance, and a greenish-yellow color, very characteristic of the north and northeast regions of the states of Paraná, São Paulo, and Minas Gerais [31–33]. The PRP used had all suitable characteristics to be used in the following experiments.

### Preparation of mold

When using polydimethylsiloxane (PDMS), the combination of base and catalyst resulted in a translucent mixture, which was easy to handle. However, there was intense formation of air bubbles. In order to reduce the bubbles in the final mold, the flask containing the mixture was placed in an ultrasonic bath and, subsequently, in a vacuum desiccator, until all bubbles were removed. With the mixture being completely clear, it was possible to insert the master structure for mold development (Supporting Information File 1, Figure S1). After 24 h, after the careful removal of the cartridge, a flexible mold was obtained, with intact and visible micropores, both on its surface and on its extension.

### Fabrication of microneedles

After obtaining the 18 formulations, all were analyzed regarding structure integrity, formation of the 36 microneedles per patch, presence of air bubbles, and also the facility of removing from the mold. The MNs containing ethanolic extract of propolis (EE) showed more integrity and were easier to be removed from the mold. MNs containing glycolic extract (GE) were more difficult to remove from the mold, as there was an increase in the concentration of extract. The formulations containing 8% (w/w) GE did not result in MNs with good integrity, due to the high amount of propylene glycol, which yielded more malleable structures. Previous studies have shown that films containing PVA and propylene glycol are more malleable due to the breaking of hydrogen bonds by the effect of propylene glycol [34].

The factorial design chosen allowed us to evaluate the characteristics of the formulations containing different concentrations of EE and EG in addition to the concentration of P407. This made it possible to choose the best concentrations for each type of extract.

In previous studies carried out by Bruschi and colleagues, the concentrations of PRP extract that would have biological activity were studied. The ranges and concentrations used in this study were chosen so that the formulation obtained has a sufficient amount of PRP extract for biological activity [31,35–36].

### Morphological analysis

**Figure 1:**
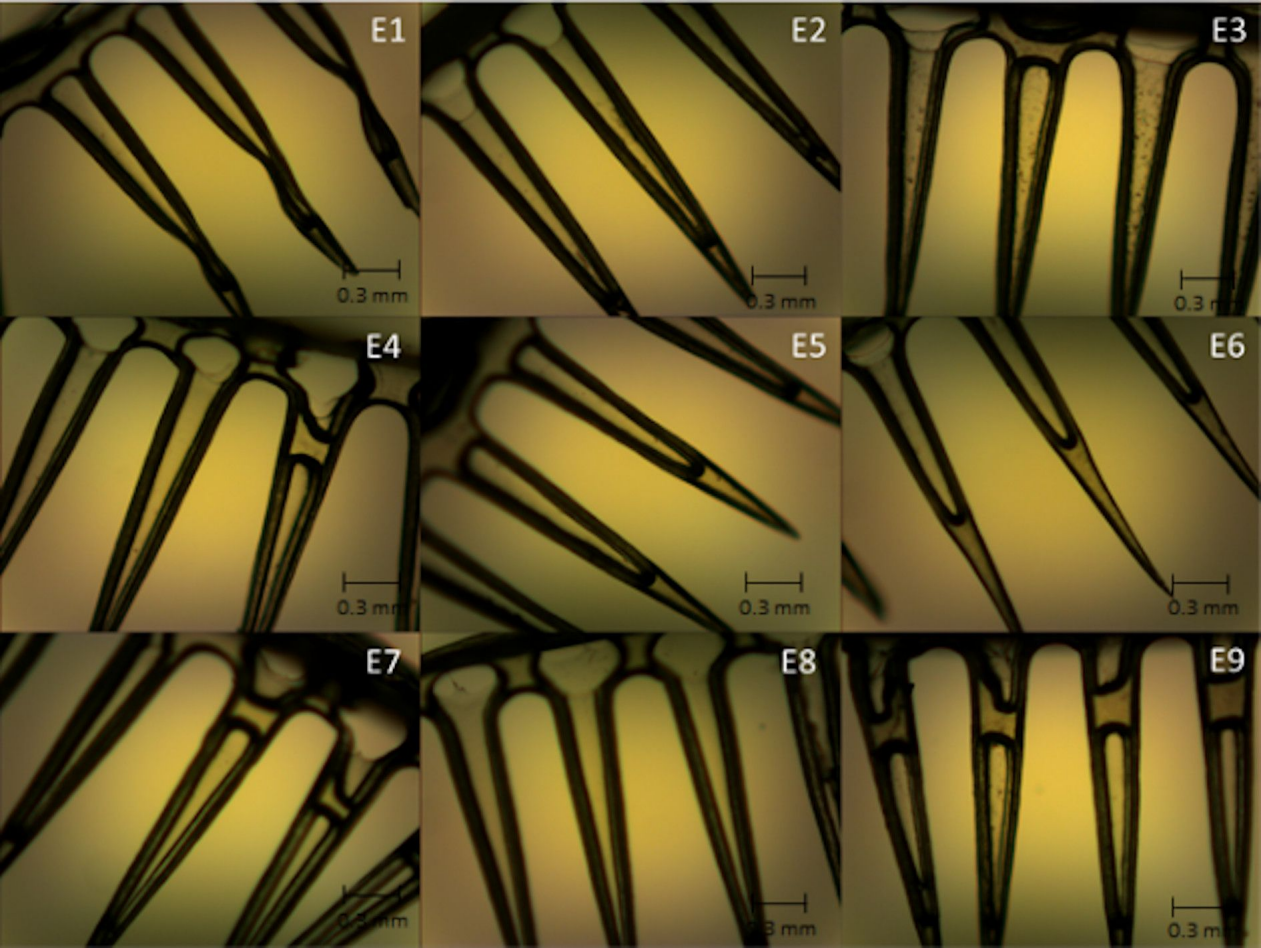
Micrographs obtained by optical microscopy showing the structure of the microneedles containing ethanolic extract of propolis (E1 to E9); magnification 40×.

**Figure 2:**
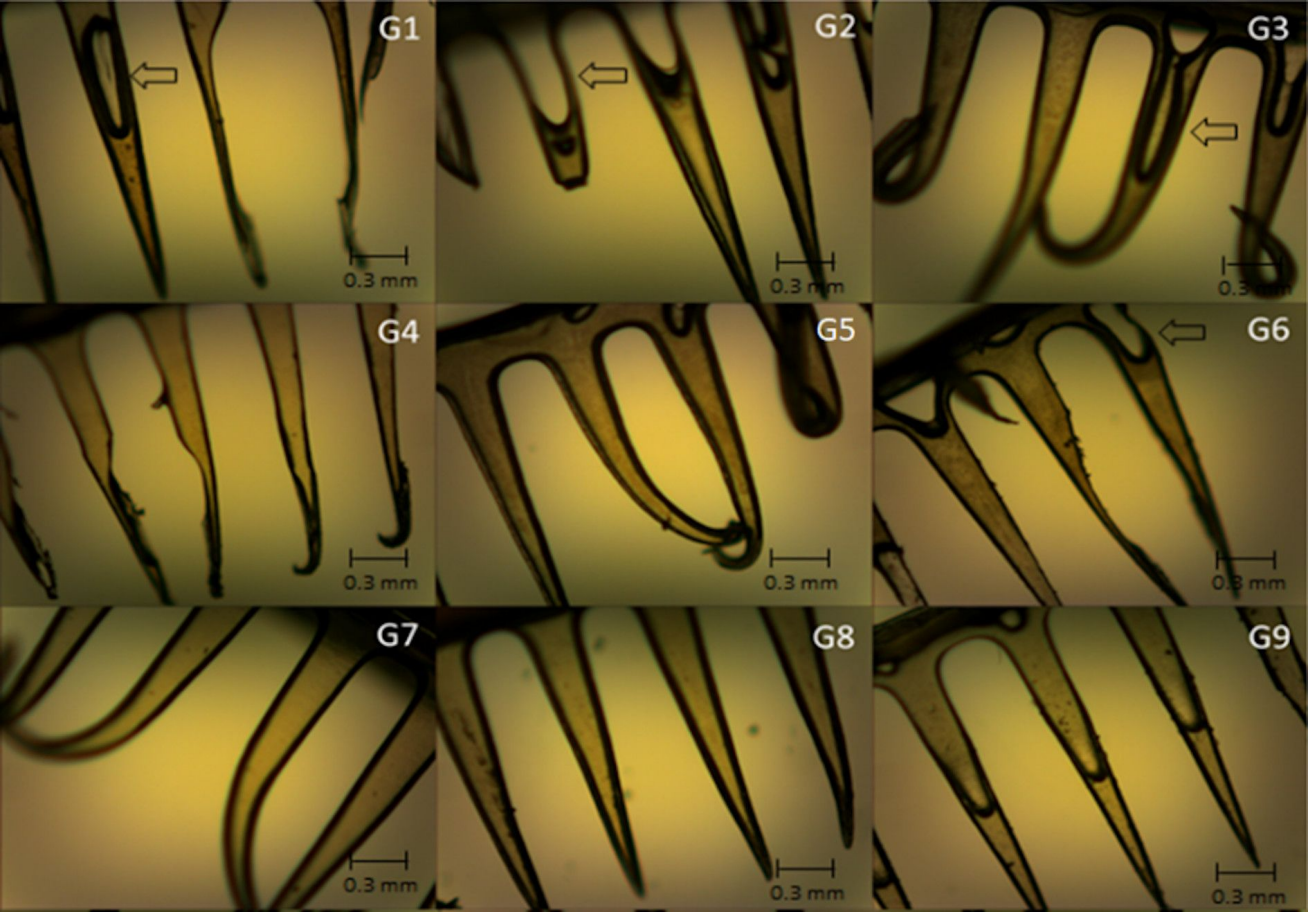
Micrographs obtained by optical microscopy showing the structure of microneedles containing glycolic extract of propolis (G1 to G9); magnification 40×. Arrows represent locations where bubbles are present.

The formulations could successfully replicate the master mold shape (Supporting Information File 1, Figure S2), and the obtained structures were macroscopically analyzed. Patches composed of 36 MNs with a spherical base and sharp tips were obtained for the majority of the formulations, mainly for those containing EE. All formulations were evaluated by optical and scanning electron microscopy for a better visualization of their structure. The micrographs obtained by optical microscopy for the all formulations are displayed in Figure 1 and Figure 2.

The micrographs show differences between MNs containing EE and those containing GE. The presence of EE resulted in MNs being more structured and firmer; however, from the images it can be seen that inside of each needle there are air bubbles or less deposits of extract. The MNs containing GE showed to be more malleable; they were more homogeneous in the dispersion of the extract; however, they also displayed air bubbles along the structure.

**Figure 3:**
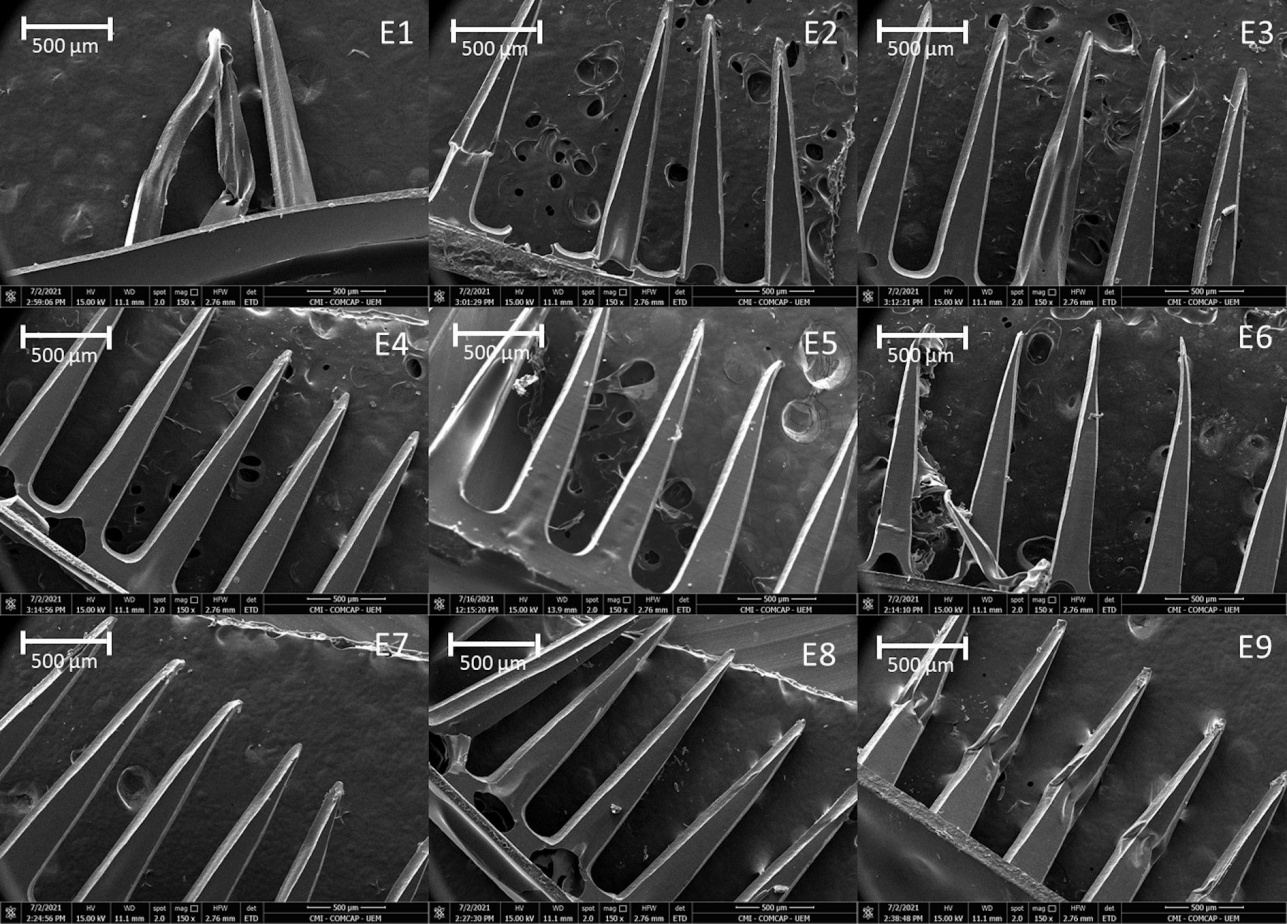
Micrographs obtained by scanning electron microscopy (SEM) showing the surface morphology microneedles containing ethanolic extract of propolis (E1 to E9); magnification 150×.

**Figure 4:**
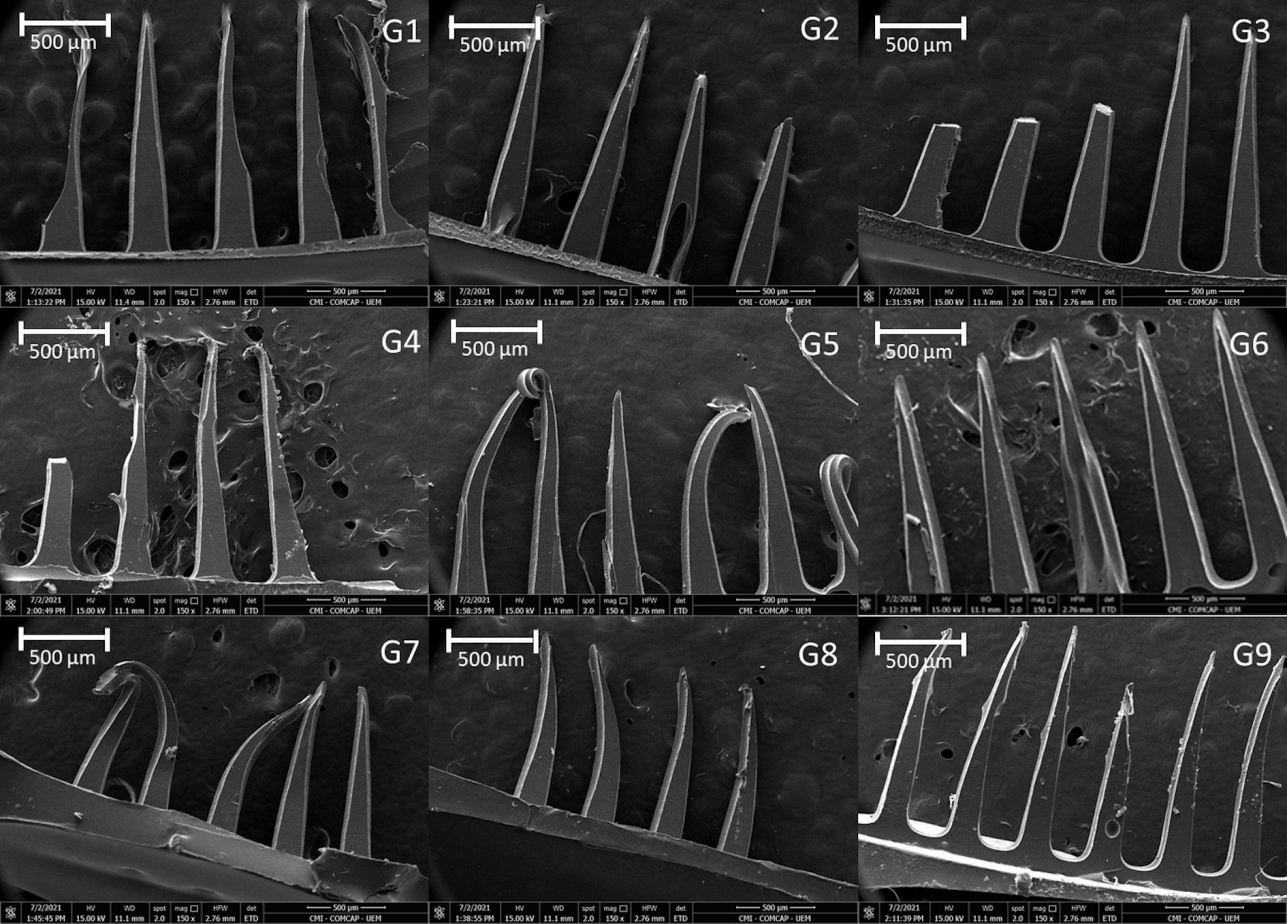
Micrograph obtained by scanning electron microscopy (SEM) showing the surface morphology of microneedles containing glycolic extract of propolis (G1 to G9); magnification 150×.

The morphological characteristics of the preparations were evaluated by SEM (Figure 3 and Figure 4), which confirmed the uniform and regular morphology of the needles. Micrographs of the formulations revealed polymeric fragments with well-defined, homogeneous structures, showing that the preparation used to obtain the structures was effective.

Micrographs obtained by scanning electron microscopy (Figure 3 and Figure 4) corroborate the results obtained from optical microscopy (Figure 1 and Figure 2), MNs containing EE demonstrated better structuring and MNs with GE demonstrated to be more malleable, with some being not formed at all (e.g., compare MNs E5 and G5, where the MNs in E5 are straight and without deformations, while the MNs in GE 5 have a malleable structure).

All formulations were analyzed regarding size. Base and height of MNs were determined to confirm the complete formation of each needle considering the total entry into the mold and characterized the size of the obtaining MNs. The measurements were taken from one of the 36 needles present in the patch (as shown below in Figure 7). The results of measurements for all MNs are shown in Table 1.

**Table 1:** Size analysis of microneedles containing ethanolic (EE) or glycolic extract (GE) of propolis.

EE			GE		

Formulation	Height (mm)	Base (mm)	Formulation	Height (mm)	Base (mm)

E1	1.6896 ± 0.0835	0.3471 ± 0.0029	G1	1.8310 ± 0.0305	0.3046 ± 0.0036
E2	1.8220 ± 0.0398	0.3109 ± 0.0080	G2	1.4469 ± 0.4359	0.3290 ± 0.0088
E3	1.6402 ± 0.0074	0.3262 ± 0.0018	G3	1.3802 ± 0.2444	0.2978 ± 0.0094
E4	1.6483 ± 0.0167	0.3216 ± 0.0151	G4	1.5806 ± 0.0173	0.2786 ± 0.0049
E5	1.7946 ± 0.0193	0.3125 ± 0.0079	G5	1.4446 ± 0.2019	0.3097 ± 0.0191
E6	1.9248 ± 0.0269	0.3174 ± 0.0119	G6	1.4007 ± 0.3192	0.2671 ± 0.0150
E7	1.9182 ± 0.0362	0.3419 ± 0.0134	G7	1.6577 ± 0.2206	0.3357 ± 0.0193
E8	1.8138 ± 0.0110	0.3279 ± 0.0117	G8	1.6384 ± 0.0454	0.3304 ± 0.0096
E9	1.7891 ± 0.0249	0.3418 ± 0.0022	G9	1.6279 ± 0.1809	0.2910 ± 0.0115

MNs containing EE were 1.78 ± 0.11 mm in height and 0.33 ± 0.01 mm in width of the base. The MNs containing GE were 1.56 ± 0.15 mm in height and 0.30 ± 0.02 mm in width of the base with smaller proportions due to the solvent affecting the formation of structures. There is a decrease of 10% and 22% in MN height, when EE and GE, respectively, are compared with the depth of the master mold. This is related to solvent evaporation as described in the literature for other biopolymeric MNs prepared by solvent casting [37–38]. The above sizes allow the MNs to rupture the stratum corneum but not reach the blood vessels, creating ducts that facilitate the flow of large molecules, nanoparticles, and proteins through the skin [39–40].

Considering the height, the MNs containing EE and P407 exhibited a significant positive interference (*p* > 0.05) (Supporting Information File 1, Table S2 and Figure S5), being greater for the amount of extract, which corroborates the results that MNs containing more EE were better formed macroscopically. As for GE, none of the variables significantly interfered in the height measurement (*p* > 0.05), which was confirmed by the difficult formation of MNs containing GE. It can also be confirmed by the response surface graphs; however, greater height values were observed for smaller amounts of P407.

Regarding the measurement of the base width, the amounts of both extracts presented significant negative interference in the final value (*p* < 0.05) (Supporting Information File 1, Table S2 and Figure S5). For GE, positive interference was observed, and for P407 and EE, negative interference was observed. Positive interferences mean that an increase of the studied condition generates an increase in the response, negative interferences mean a decrease of the response with an increase of the studied condition [41], in this case the concentration of PRP and P407 extract. Even with relatively low *r* values, it was possible to observe the behavior of the formulations with different amounts of PRP and P407 extract regarding height and base width measurements. The base width values of all formulations were quite similar, mainly due to the standardized use of molds.

**Figure 5:**
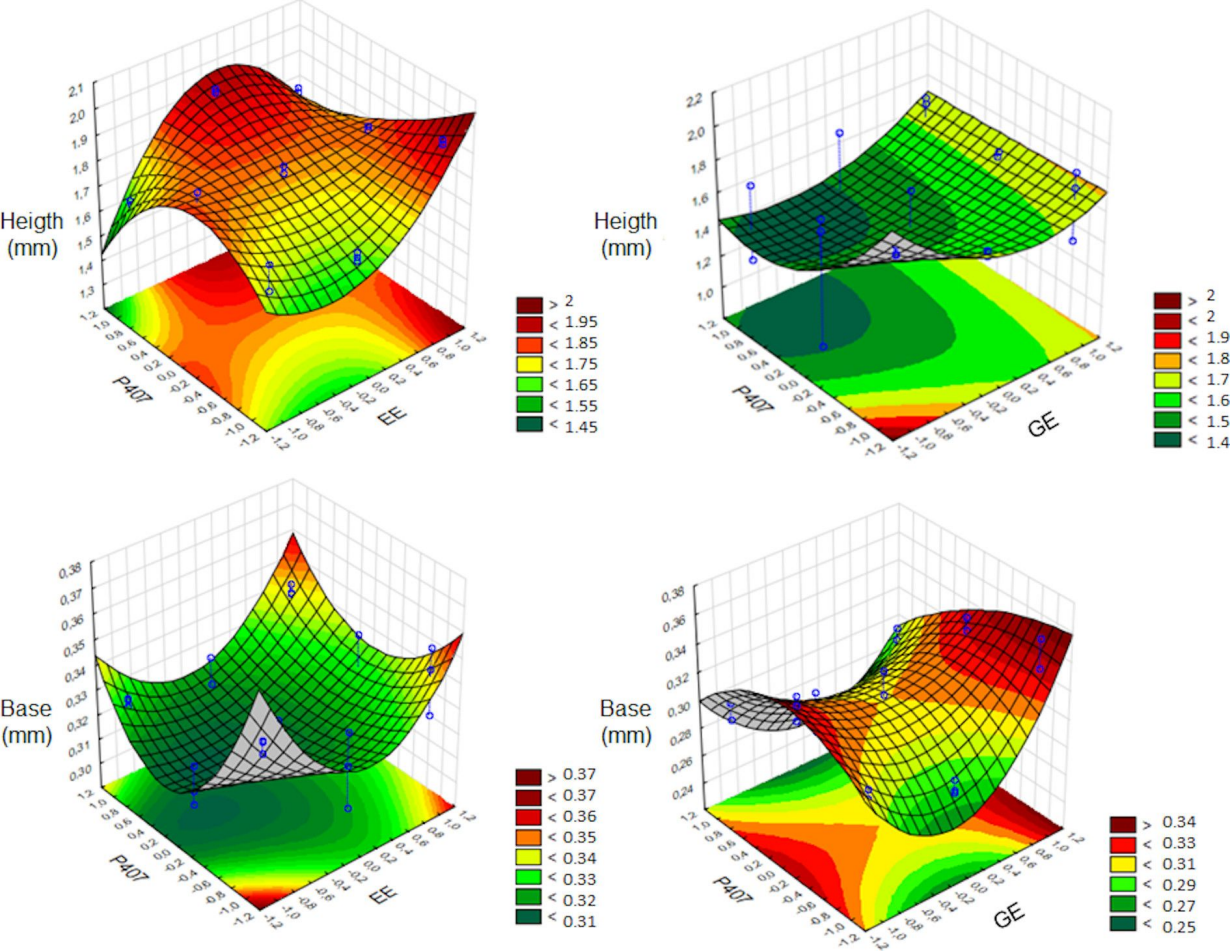
Response surface plots for height and base measurements of MNs containing EE or GE. The color scale is indicated in each figure and shows the isoparametric values.

The response surface is an advanced experiment design technique that helps to understand and optimize the best response. This tool is used to develop a functional relationship between a response of interest, and also to refine models after determining the important factors by selecting the performed experiments [41]. The response surfaces for analysis of height and base width measurements are shown in Figure 5.

The equations obtained for the height and base measurement responses, as well as the values for the correlation coefficient (*r*), from the statistical analysis of the results obtained for the measurements of microneedles are given in Table 2.

**Table 2:** Equations obtained for the height and base responses with the correlation coefficient *r*.

	EE		GE	

	Equation	*r*	Equation	*r*

Height	*Y* = 1.7823 + 0.1231*X*_1_ − 0.0985*X*_1_·*X*_2_ + 0.0417*X*_22_²	0.9167	*Y* = 1.5565	0.3583
Base	*Y* = 0.3275 − 0.0155*X*_11_^2^ – 0.0156*X*_22_^2^	0.7218	*Y* = 0.3049 − 0.0210*X*_2_ − 0.0189*X*_1_·*X*_2_ − 0.0296*X*_11_^2^ + 0.0272*X*_22_^2^	0.8316

As the base width/height ratio decreased, the failure force decreased as well. This can be observed by the MNs E5, which have the lowest compression force of the MNs containing EE and an aspect ratio of 1:7 (base width/height) while the MNs E9 have the highest compression value and an aspect ratio of 1:6 (base width/height). Römgens and colleagues also observed that with an increase in the diameter of the MN tips, the force for insertion increases [42]. When different MN tip diameters were studied for insertion into the biological substrate, it was observed that MNs with a diameter of 5 µm presented greater penetration power than MNs with larger diameters [42]. The MNs containing EE and GE displayed similar tip shapes and sizes as the MNs of this previous study, thus favoring penetration.

### Analysis of hardness

The 18 formulations were analyzed for compression force and compression area on a hard glass surface (Petri dish). The results are displayed in Table 3.

**Table 3:** Compression force and compression area of MNs containing EE or GE on a hard glass surface. Each analysis was performed, at least, in triplicate.

Formulation	Compression force (N)	Compression area (N·mm)	Formulation	Compression force (N)	Compression area (N·mm)

E1	0.6133 ± 0.0984	7.9167 ± 1.2208	G1	0.3075 ± 0.0170	2.5867 ± 0.2097
E2	0.6034 ± 0.0489	10.9777 ± 4.0329	G2	0.2220 ± 0.0233	2.4287 ± 0.5104
E3	0.6475 ± 0.0493	7.6293 ± 1.5393	G3	0.4153 ± 0.1330	4.9460 ± 1.9119
E4	0.3436 ± 0.0383	2.4487 ± 0.5678	G4	0.3090 ± 0.0001	2.2160 ± 0.0001
E5	0.3392 ± 0.2011	2.4637 ± 1.5757	G5	0.6140 ± 0.1005	7.1507 ± 1.7283
E6	0.5229 ± 0.0346	4.6517 ± 1.1466	G6	0.4833 ± 0.0298	4.5703 ± 0.6339
E7	1.1130 ± 0.2569	19.0077 ± 4.9568	G7	0.2260 ± 0.0001	2.0580 ± 0.0001
E8	1.1981 ± 0.0231	18.5693 ± 3.2540	G8	0.1245 ± 0.0001	1.1800 ± 0.0001
E9	1.2345 ± 0.0138	15.9167 ± 0.4926	G9	0.2053 ± 0.0124	1.4380 ± 0.1895

Regarding the compression force, for MNs containing EE, only the extract showed positive significant interference, where higher values of compression force were observed for MNs containing a greater amount of extract, with the highest value obtained for the E9 formulation, which contains 3% P407.

**Figure 6:**
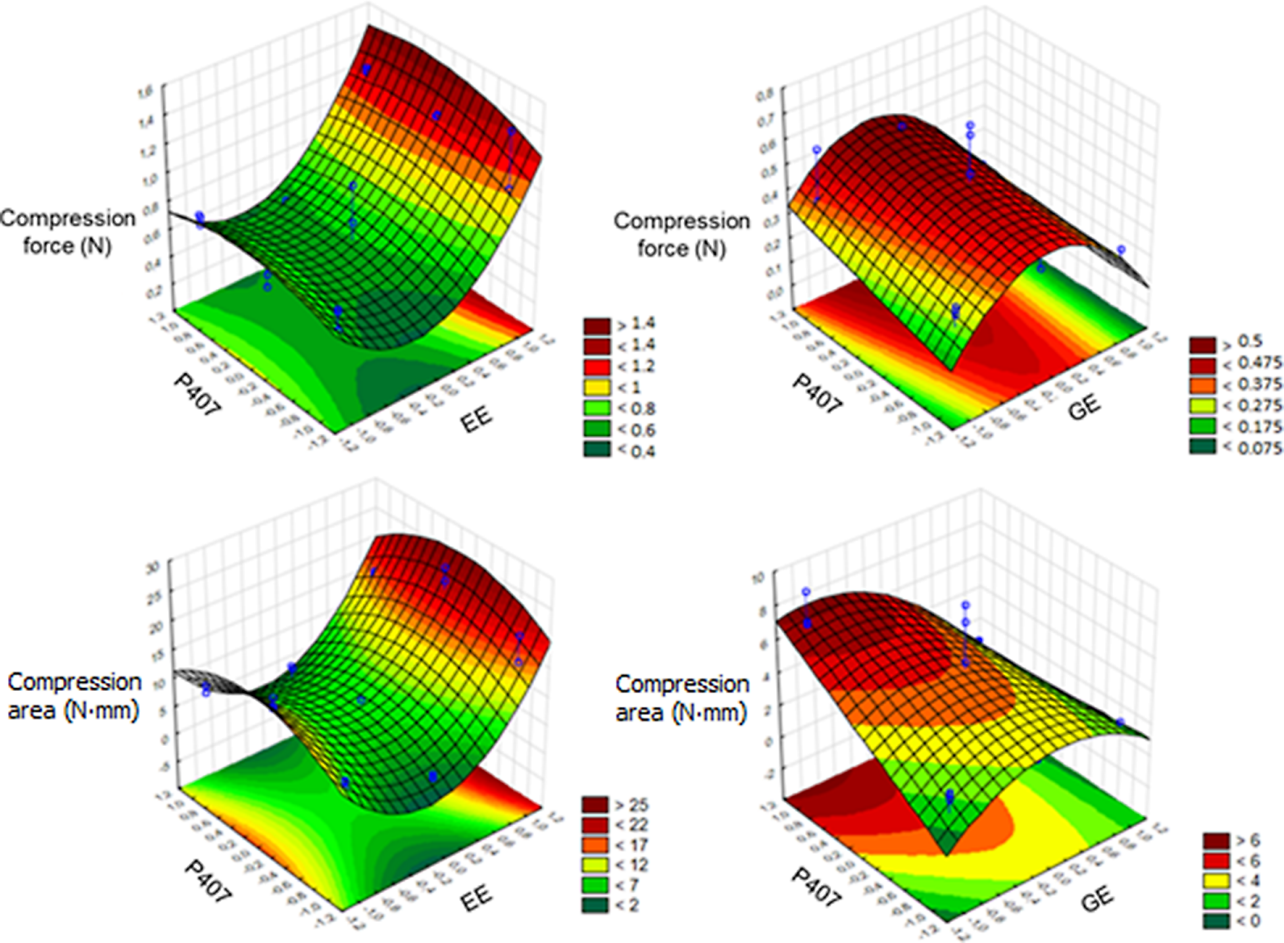
Response surface plots for force and compression area of MNs containing EE or GE. The color scale is indicated in each figure and shows the isoparametric values.

Considering MNs containing GE, both independent variables had significant interference, being negative for GE and positive for P407. The formulations that presented greater compression force were the formulations G5 and G6, containing 4% of GE and 2% and 3% of P407 respectively. The respective equations obtained were *Y* = 0.7928 + 0.5232*X*_1_ − 0.3523*X*_11_^2^ and *Y* = 0.3230 − 0.1297*X*_1_ + 0.0871*X*_2_ + 0.2187*X*_11_^2^ for MNs containing EE or GE, respectively. The response surface plots for analysis of force and compression area are displayed in Figure 6.

For the compression area of MNs containing EE, the highest values were observed for the formulations with 12% of EE, whereas for the formulations with GE the highest values were observed for the formulations containing 4% of GE. The respective equations obtained were *Y* = 11.1227 + 7.3499*X*_1_ − 9.4281*X*_11_^2^ + 4.0988*X*_22_^2^ and *Y* = 3.5036 − 2.6528*X*_1_ + 2.3812*X*_2_ − 2.8720*X*_1_·*X*_2_ + 1.8557*X*_11_^2^. The results of strength and compression area were essential for choosing the best formulations, in addition to showing the best structures for the amounts of extract and P407 used.

In previous studies of Donnelly and colleagues [4], MNs composed of PVA, alginic acid, Carbopol 971P, and Gantrez were synthesized and also mechanically analyzed. It was observed that, for values greater than 0.03 N per needle, it would already be possible to perforate the stratum corneum to release the active substance. In comparison with the results obtained, formulations E7, E8, and E9 would have presented compression forces in a petri dish sufficient to perforate the initial layer of the skin. In Supporting Information File 1, Figure S6 it is possible to observe the structure of the MNs before and after the compression test.

Afterwards, the best formulations were chosen to proceed with further characterizations. The selected formulations were E3, E6, E9, and G6. They are composed of 3% P407 and 4%, 8%, and 12% EE, or 4% GE, respectively. These formulations were chosen because they exhibited macroscopic integrity and the formulations containing EE with 3% of P407 presented the highest values in the mechanical analysis. Although the MNs containing GE were malleable, the formulation GE6 was chosen because it showed good integrity, with values similar to those of E3. It was utilized for comparison with the formulations containing EE.

### Compression test on different surfaces

The best formulations (E3, E6, E9, and G6) were mechanically evaluated in compression tests on PVC film, Parafilm M, gelatin, and porcine skin. The results for the compression force of the best formulations are displayed in Table 4. The MNs did not yield total penetration of PVC film, Parafilm M, and porcine skin; however, it was verified that the applied force was sufficient to mark the substrates. All evaluated formulations displayed penetration of the gelatin substrate, including the MNs without PRP extract and the stainless-steel MNs (standard) used to obtain the mold. Previous studies have shown that MNs composed of Gantrez S-97 and PEG 10,000 had differences when inserted into porcine skin and layered Parafilm M [43].

**Table 4:** Compression force (N) of the selected MN formulations applied on different surfaces (PVC film, Parafilm M, gelatin and porcine skin). Each analysis was performed, at least, in triplicate.

Formulations	Force (N)			

	PVC film	Parafilm M	Gelatin	Porcine skin

E3	0.4247 ± 0.0123	0.3863 ± 0.0090	0.1479 ± 0.0129	0.0979 ± 0.0017
E6	0.5085 ± 0.0116	0.6319 ± 0.0253	0.2726 ± 0.0094	0.1431 ± 0.0093
E9	0.5492 ± 0.0161	0.7476± 0.0394	0.2737 ± 0.0120	0.1438 ± 0.0055
G6	0.3989 ± 0.0031	0.3422 ± 0.0164	0.2231 ± 0.0103	0.0866 ± 0.0064
MNs without propolis extract	0.1369 ± 0.0099	0.3429 ± 0.0857	0.1030 ± 0.0041	0.1893 ± 0.0158
Stainless still MNs (standard model)	0.1543 ± 0.0109	0.7943 ± 0.0525	0.3589 ± 0.0108	0.2406 ± 0.0231

For all analyses, as the amount of extract in the formulations increases, the compression force also increases regardless of the surface utilized in the test. The formulation G6 displayed the lowest results, probably due to the presence of propylene glycol in the formulation. Considering that the selected formulations contained 3% P407, the amount of PRP extract was the only factor that influenced the increase in the compression force. Thus, it can be inferred that the hardness of MNs increases with the increase in the concentration of PRP extract in the formulation. MNs with GE were malleable due to the plasticizing effect of propylene glycol, which resulted in lower mechanical strength [34]. In contrast, the presence of EE resulted in better structured MNs that were harder and had greater penetration capacity.

In other studies using MNs composed of PVA, Carbopol 971P, and alginic acid, it was observed that with a force of 0.03 N or greater, most MNs could penetrate the stratum corneum [4]. Thus, the force values displayed for the selected formulations also indicate the ability to penetrate porcine skin.

All selected MN formulations were statistically compared with MNs without PRP extract and stainless-steel MNs (standard) using Students’ *t*-test evaluation of possible significant differences among the compression forces for each substrate/surface utilized. The results for the *p* values are displayed in Supporting Information File 1, Table S1. There were no statistically significant differences for PVC film when the formulations E3 and G6 were compared with the formulations without extract and the standard. For Parafilm M, no significant differences were observed when comparing E3 and MNs without PRP, G6 and MNs without PRP, and MNs without PRP and the standard. For the analysis using gelatin as surface, only the difference between formulations E6 and E9 was not statistically significant. When the surface was porcine skin, the differences between E6 and E9, E3 and G6, and MNs without PRP and standard were not statistically significant (Figure S3, Supporting Information File 1).

Supporting Information File 1, Figure S4 displays images of the different surfaces (PVC film, Parafilm M, gelatin, and porcine skin) after the compression test with MN formulations. It was not possible to observe any punctures or, consequently, to stain the porcine skin, possibly due to the thickness of the skin.

## Conclusion

The present study demonstrated, for the first time, the design of MNs composed of PVA, PVP, and P407 as a polymeric platform for the delivery of alcoholic and glycolic green propolis extracts. MN formulations were characterized regarding their morphology, dimensions, and mechanical properties. Selected MN formulations (E3, E6, E9, and G6) exhibited suitable mechanical strength to penetrate different substrates that mimic the human skin, making them potentially useful systems for topical propolis administration. As MNs are minimally invasive platforms, the selected systems can be easy to apply and monitor. Further studies should be conducted regarding in vitro and in vivo evaluation of the biological activity and cytotoxicity of the MNs, the vitro PRP release profile, the cutaneous permeation using porcine and human skin, and photoacoustic spectroscopy.

## Experimental

### Materials

Poloxamer 407 (P407), gelatin, and propylene glycol were purchased from Sigma (St. Louis, MO, USA). Polyvinyl alcohol (PVA) was obtained from Neon (Sao Paulo, SP, Brazil), polyvinylpyrrolidone (PVP), and ethanol 96° GL was purchased from Labsynth (Sao Paulo, SP, Brazil). Sylgard^TM^ 184 silicone elastomers kit (polydimethylsiloxane; PDMS) was purchased from Dow (Midland, MI, USA). Purified water was obtained from a water purification system (Evoqua Water Technologies, Pittsburgh, PA, USA). Absolute ethanol was purchased from Anidrol (Sao Paulo, SP, Brazil).

### Preparation and characterization of PRP extracts

Brazilian green propolis (PRP) was obtained from an apiary of *Apis mellifera* L. bees, located inside a eucalyptus reserve, surrounded by native forest with predominance of *Baccharis dracunculifolia* (Asteraceae), in the northwest of Parana state. This research was registered in Brazil with SISGEN N° AC7A2F5. The different PRP extracts were prepared by turbo extraction. The ethanolic extract (EE) was obtained using ethanol 96° GL and the comminuted drug with a drug/solvent (w/w) ratio of 3:7. The glycolic extract (GE) was prepared using the comminuted drug and an aqueous solution of propylene glycol 50% (w/w) and the same drug/solvent (w/w) ratio of 3:7. The final dispersions were filtered through grade-3 filter paper [44–45].

The pH determination was performed using a pHmeter calibrated with buffer solutions. The relative density of the extract was determined using a pycnometer calibrated at 20 °C in a controlled temperature environment (20 °C). To determine the dry residue, exactly 1 g of extract was weighed, the excess solvents were evaporated on a hot plate for approximately 10 min. Subsequently, the containers containing the extracts were taken to an oven (110 °C) for 40 min until constant weight. An aliquot of 12.5 mL of the extractive solution was placed in a round-bottomed flask, added to 50.0 mL of purified water and subjected to simple distillation. The distillate was collected in a 50.0 mL volumetric flask and made up to volume with purified water. The distillate density was determined, and from the density the alcohol content was determined.

The determination of the total polyphenol content was performed in 25.0 mL volumetric flasks, 10 mL of purified water were added and 10.0 μL of extract were added. Then, 1.0 mL of phosphomolybdotungstic reagent R (Folin–Ciocalteau) was added and the volume was made up with 14.06% (m/V) aqueous sodium carbonate solution. The extracts were analyzed using a high-performance liquid chromatograph coupled to a UV–vis spectrophotometric detector. For the detection of polyphenols, chrysin and p-coumaric acid (analytical standard) at a wavelength (λ) of 310 nm were used as markers. At least three determinations were performed for each assay [36,46–48].

### Preparation of silicone molds

The master structure utilized to fabricate the molds was a stainless-steel microneedle system (cartridge) from DermaPen (Belrose, Australia) containing 36 microneedles of 2 mm in length. The molds developed were inverse replicas of this master structure.

For the preparation of the PDMS mold, suitable amounts of the catalyst agent and the silicone base were mixed, in a proportion of 1:6, using a beaker and taken to ultrasound for 5 min. Afterwards, the mixture was poured into 12-well plates and taken to ultrasound for another 5 min. The microneedle cartridge was inserted into the mixture and placed under ultrasound for 5 min to remove all air bubbles. The plate containing the silicone and the microneedle cartridge was placed in a vacuum desiccator for silicone curing. After 24 h, the cartridge was carefully removed and the mold obtained was cleaned and evaluated for integrity for later use.

### Fabrication of microneedles

Polymeric MNs were prepared using the mold casting technique. The polymeric matrix, composed of PVA, PVP, P407, and the different types of PRP extract, was prepared to compose the microneedle system. For the development of MNs, three different concentrations of P407 and PRP extract were used in a fixed matrix of PVA/PVP using a ratio of 1:1. P407 was used at 1%, 2%, and 3% (w/w) and PRP extract at 4%, 8%, and 12% (w/w) for the ethanolic extract and 2%, 4%, and 8% (w/w) for the glycolic extract, resulting in a factorial design of 3^2^, totaling nine formulations for each type of extract, rendering a total of 18 formulations (Table 5).

**Table 5:** Experimental 3^2^ design utilized for the development of microneedles (MNs) composed of polyvinyl alcohol (PVA) and polyvinylpyrrolidone (PVP) in a fixed 1:1 ratio, poloxamer 407 (P407), and ethanolic propolis extract (EE) or glycolic propolis extract (GE).

Independent variables	Levels		

	Low (−1)	Central (0)	High (+1)

*X*_1_ = P407 (%, w/w)	1	2	3
*X*_2_ = EE (%, w/w)^a^	4	8	12
*X*_2_ = GE (%, w/w)^b^	2	4	8

Standard run (formulations)	*X*_1_	*X* *_2_*	

E1	−1	−1	
E2	0	−1	
E3	+1	0	
E4	−1	0	
E5	0	+1	
E6	+1	+1	
E7	−1	−1	
E8	0	−1	
E9	+1	0	
G1	−1	0	
G2	0	+1	
G3	+1	+1	
G4	−1	−1	
G5	0	−1	
G6	+1	0	
G7	−1	0	
G8	0	+1	
G9	+1	+1	

^a^formulations containing EE (block a); ^b^formulations containing E (block b).

Firstly, each polymer was dispersed separately. To PVA, 8 mL of purified water were added, and the mixture was heated and stirred until completely dispersed. The suitable amount of PVP was dissolved in 3 mL of absolute ethyl alcohol, and P407 was dissolved in 2 mL of the same solvent. After obtaining the individual dispersions, the P407 was poured onto the PVP, magnetically stirred for 10 min, and this mixture was poured onto the PVA under magnetic stirring for another 10 min, after which the PRP extract was placed and the mixture was stirred for 20 min.

Afterwards, the final formulations were centrifuged at 3200 rpm for 10 min to remove air bubbles. Each formulation was carefully poured onto the molds to form a complete microneedle patch. Then, they were placed in a desiccator for 48 h for complete casting. Subsequently, the MNs were carefully recovered from the molds with the help of tweezers and stored in desiccators until future use.

### Experimental design

The 3^2^ full factorial design (two blocks) was utilized to determine the influence of P407 (*X*_1_) and PRP extract (*X*_2_) concentrations on height and base measurements, strength, and printing area in a hard surface (glass Petri dish) [49]. The independent factors were evaluated at three levels, low (−1), central (0) and high (+1) (Table 5). The surface response plots of the MN system behavior were utilized to show the interaction effects of the independent variables (P407 and EE or GE) on height and base measurements, strength, and printing area in the hard surface. To predict the optimal conditions, a polynomial equation was fitted correlating the relationship between the independent variables and the response:


[1] $$Y=b_{0}+b_{1}X_{1}+b_{2}X_{2}+b_{12}X_{1}X_{2}+b_{11}X_{11}{}^{2}+b_{22}X_{22}{}^{2}$$

where *Y* is the response (the dependent variable) as function of *X*_1_ and *X*_2_, *b*_0_ is a constant term (the arithmetic mean response of nine batches), *b*_1_ and *b*_2_ are the estimated coefficients of linear terms, and *b*_12_ is the coefficient of the interaction effect. The polynomial terms *X*_11_^2^ and *X*_22_^2^ consider the non-linearity, and the interaction term *X*_1_*X*_2_ shows the response changes when two factors are simultaneously changed.

The experiments were randomized to minimize the influence of unexplained variability in the responses. Residual analysis was also conducted to validate the assumptions utilized in the analysis of variance and to identify outliers. The proportion of variance explained by models obtained was given by the multiple and adjusted coefficients (*r*^2^ and *r*^2^_adj_), whereas the adequacy of the model was determined by a lack-of-fit test.

### Macroscopic evaluation

**Figure 7:**
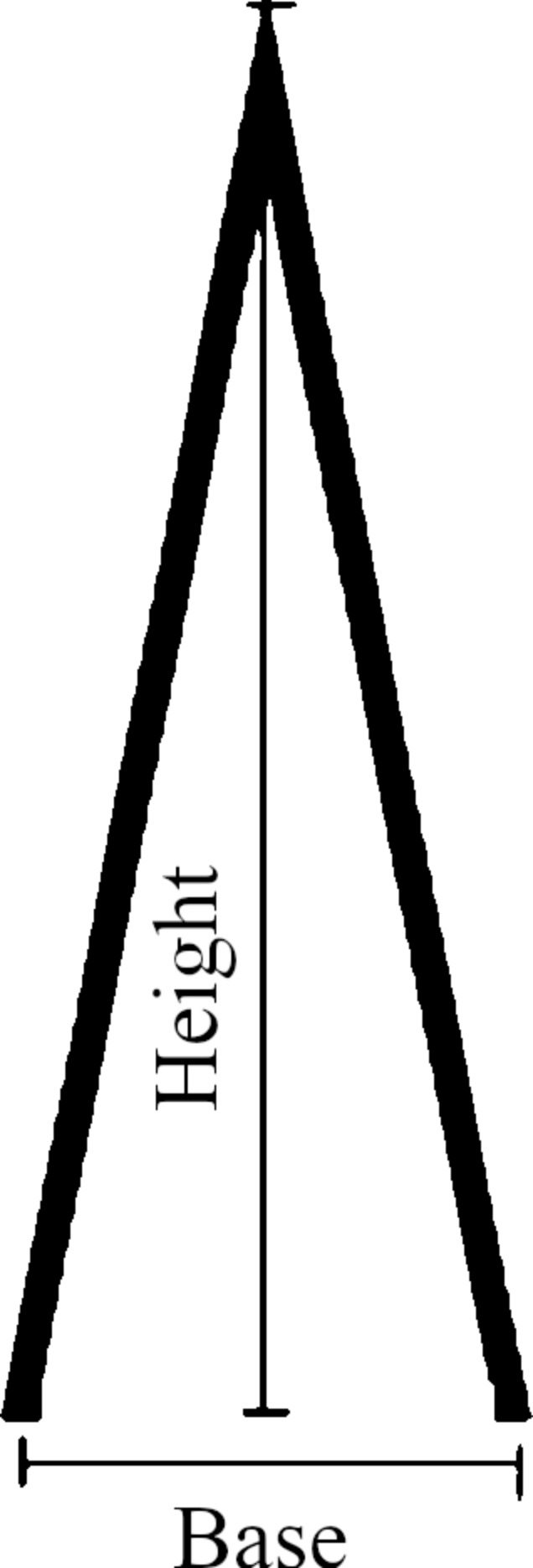
Schematic representation for the analysis of the height and base measurements of the microneedles.

The MNs obtained were evaluated macroscopically for flexibility, integrity, homogeneity, color, and presence of air bubbles. The height and base measurements of the MNs obtained were analyzed (Figure 7).

### Optical microscopy analysis

The evaluations of the morphological characteristics and dimensions of the molds and MNs were carried out by optical microscopy using an optical microscope KozoOptics (Nanjing, China), at 40× magnification. For analysis, a row of MNs was cut from the structure obtained for better visualization of the isolated microneedles.

### Scanning electron microscopy

The elucidation of more specific morphological details of the fabricated MNs was performed by scanning electron microscopy. Each sample was placed over a circular metallic holder (with the help of double-sided tape) and fixed with colloidal gold in a hermetically closed chamber in argon atmosphere. The samples were observed using a Quanta 250 electron microscope (FEI, Hillsboro, OR, USA).

### Mechanical analysis

#### Hardness

The effects of the amount of P407 and of amount and type of PRP extract on the MN hardness and mechanical resistance were quantitatively evaluated by a microneedle textural study. A compressive load was applied (i.e., force applied parallel to the vertical axis) to the polymeric MNs using a TA-XTplus texturometer (TA Instruments, Surrey, UK). The MN patches were connected to the mobile cylindrical probe (P/1 – 1″ diameter aluminium cylinder ) with double-sided tape. The probe was pressed against the defined surface (hard glass surface of Petri dish) at a rate of 0.05 mm/s. The pre-test and post-test speeds were 1 mm/s, and the applied force was 0.049 N. The length at which the MNs descended in the test was 1.5 mm. From the relationship between force and time, the compression force (the maximum force during compression) and compression area (the work required to deform the sample during compression) were calculated using the software Texture Exponent 6.1.12.0 (Stable Micro Systems, Surrey, UK).

#### Puncture

The effect of formulation composition on the puncture force and distance was evaluated by MN insertion study. The best formulations selected from previous analysis were mechanically evaluated using different surfaces. The same texturometer and probe previously described were utilized. In compression mode, the MNs patch attached to the probe was pressed against the defined surface with a force of 0.049 N at a 0.05 mm/s using 5% gelatin or porcine skin or at rate of 0.1 mm/s using PVC film or Parafilm M [4]. The pre-test and post-test speeds were 1 mm/s. The length at which the MNs descended in the test was 1.5 mm. The force required to pucture the substrate during the compression of the probe/MNs patch was calculated using the software Texture Exponent 6.1.12.0 (Stable Micro Systems, Surrey, UK). The substrate composed of an aqueous dispersion of gelatin 5% (w/w) was prepared by dispersing the weighed gelatin in warm water. After complete dispersion, the dispersion was poured into petri dishes and allowed to dry at room temperature to be used in further tests. The porcine skin samples were obtained by dissection of skins from the ears of pigs (albinos and youngsters), which were recently slaughtered. The skin patches were washed, the hairs cut and, later, the patches were dissected in order to extract the epidermis and dermis. They were cut into standardized sizes, wrapped in plastic film and aluminum foil, and kept in a freezer. At the time of use, they were thawed at room temperature, and each skin fragment was then placed in a base for the test.

### Statistical analysis

The effects of the amount of P407 and of amount and type of PRP extract on the height and base measurements, strength, and printing area in the petri dish were statistically evaluated using three-way analysis of variance (ANOVA). Tukey’s post-hoc test was used to analyze significant differences. A significance level of *p* < 0.05 was considered and the Statistica 12.0 software (StatSoft Company, Tulsa, OK, USA) was used for all analyses.

## Supporting Information

Supporting Information features pictures of molds and macroscopic appearance of the MNs, statistical analysis of the compression force of MNs applied on different substrates (PVC film, Parafilm M, Gelatin, and porcine skin), images of substrates after puncture test, statistical analysis of MN height and base measurements, and images of the MNs before and after the compression test.

File 1 Additional figures and tables.
