# Supplementary material for: Design and characterization of polymeric microneedles containing extracts of Brazilian green propolis
Source: Beilstein J Nanotechnol. 2022 Jun 8;13:503–16. doi: 10.3762/bjnano.13.42 (PMC9194495; doi:10.3762/bjnano.13.42)
Supplement: File 1 — Additional figures and tables. [file Beilstein_J_Nanotechnol-13-503-s001.pdf]

BEILSTEIN JOURNAL OF NANOTECHNOLOGY

Supporting Information

for

Design and characterization of polymeric microneedles containing extracts of Brazilian green propolis

Camila Felix Vecchi, Rafaela Said dos Santos, Jéssica Bassi da Silva and Marcos Luciano Bruschi

Beilstein J. Nanotechnol. **2022,** *13,* 503–516. doi:10.3762/bjnano.13.42

Additional figures and tables

License and Terms: This is a supporting information file under the terms of the Creative Commons Attribution License (https://creativecommons.org/licenses/by/4.0). Please note that the reuse, redistribution and reproduction in particular requires that the author(s) and source are credited and that individual graphics may be subject to special legal provisions.
The license is subject to the *Beilstein Journal of Nanotechnology* terms and conditions: (https://www.beilstein-journals.org/bjnano/terms)

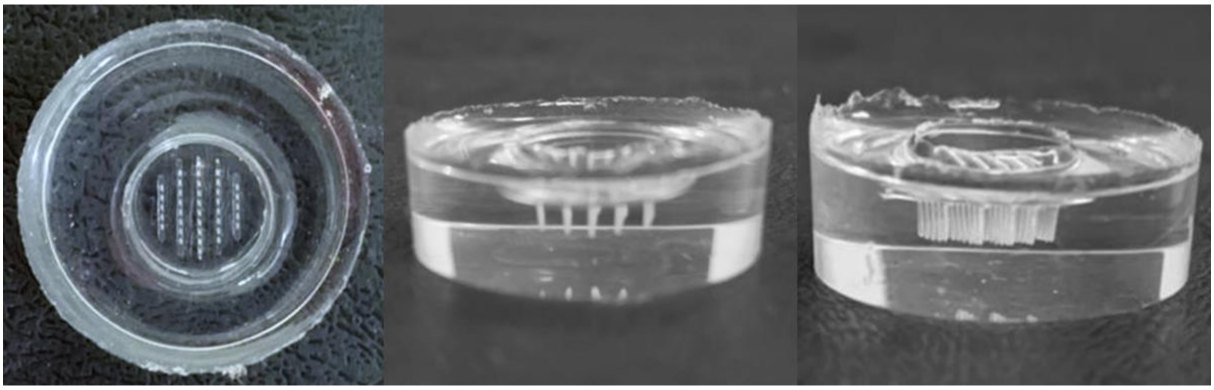

Figure S1: Photographs of the polydimethylsiloxane (PDMS) mold utilized for the fabrication of microneedles.

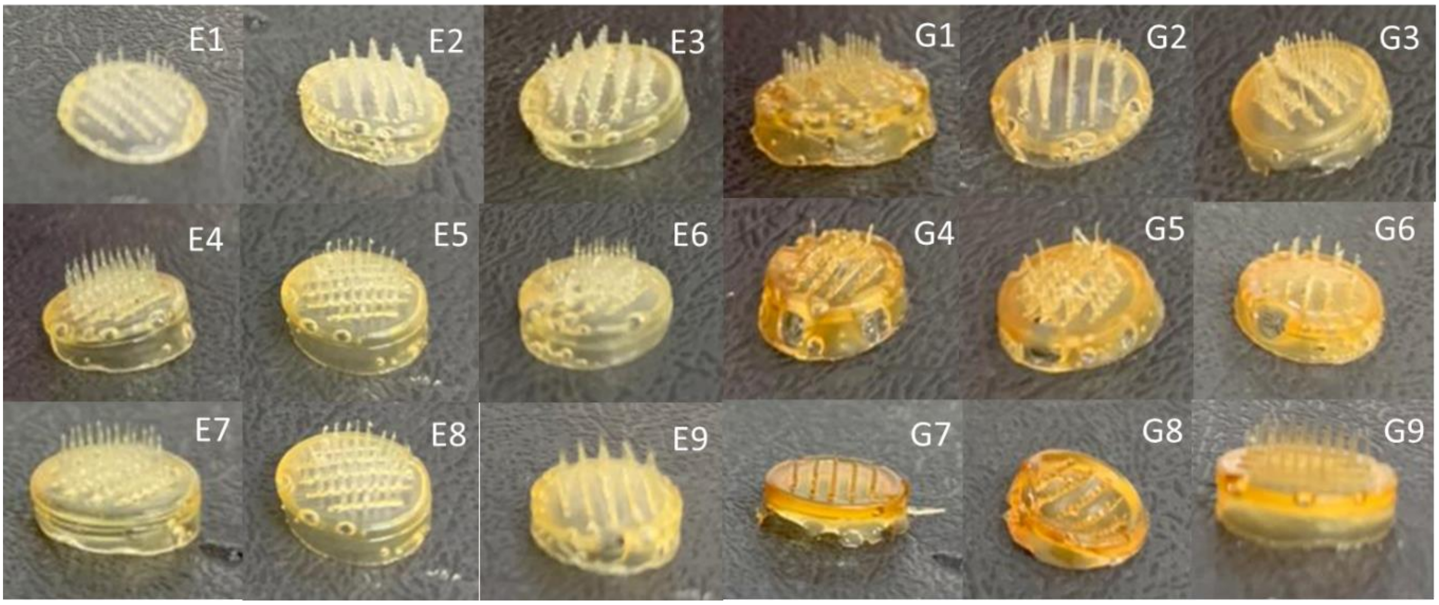

Figure S2: General aspect (macroscopic characteristics) of the microneedles containing ethanolic extract of propolis (E1–E9) or glycolic extract of propolis (G1–G9).

Table S1: Statistical values of *p* from the compressive force analysis of microneedles applied on different surfaces (PVC film, Parafilm M, gelatin, or porcine skin).

Student t test analysis		*p*-value			
Compared formulations		PVC film	Parafilm M	Gelatin	Porcine skin
E3	E6	0.0010	0.0014	0.0002	0.0119
E3	E9	0.0006	0.0026	0.0002	0.0025
E6	E9	0.0277	0.0180	0.9043	0.9175
E3	G6	0.0599	0.0247	0.0016	0.0835
E3	MN without PRP	0.0000	0.4727	0.0186	0.0091
E6	MN without PRP	0.0000	0.0210	0.0001	0.0187
E9	MN without PRP	0.0000	0.0063	0.0006	0.0272
G6	MN without PRP	0.0001	0.9910	0.0007	0.0032
E3	Standard model MN	0.0000	0.0045	0.0000	0.0083
E6	Standard model MN	0.0000	0.0186	0.0005	0.0097
E9	Standard model MN	0.0000	0.2907	0.0008	0.0145
G6	Standard model MN	0.0002	0.0023	0.0000	0.0047
MN without PRP	Standard model MN	0.1097	0.0030	0.0001	0.0399

E = microneedles containing ethanolic extract of propolis; G = microneedles containing glycolic extract of propolis; MN = microneedles; PRP = propolis

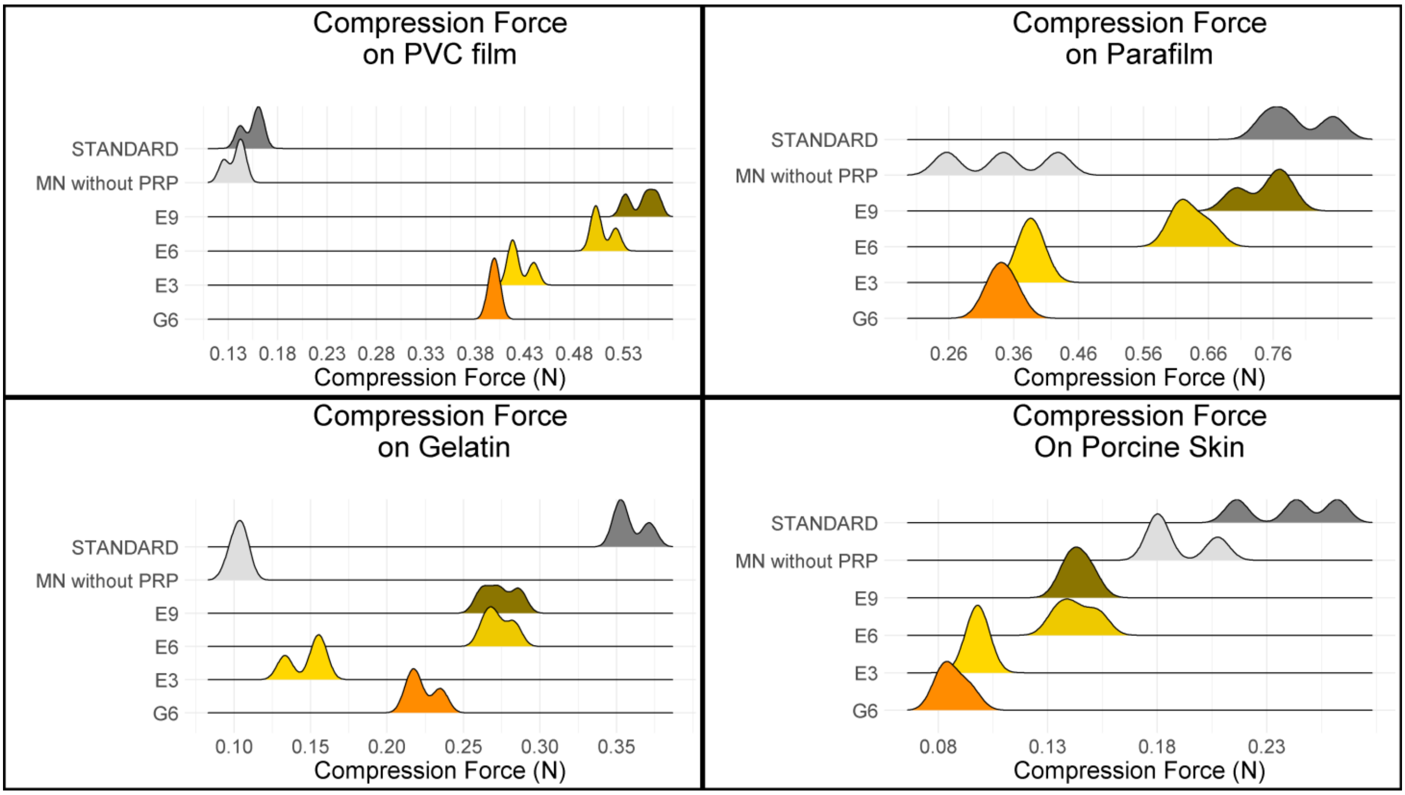

Figure S3: Statistical comparison between means by Student's t test for the analysis of puncture.

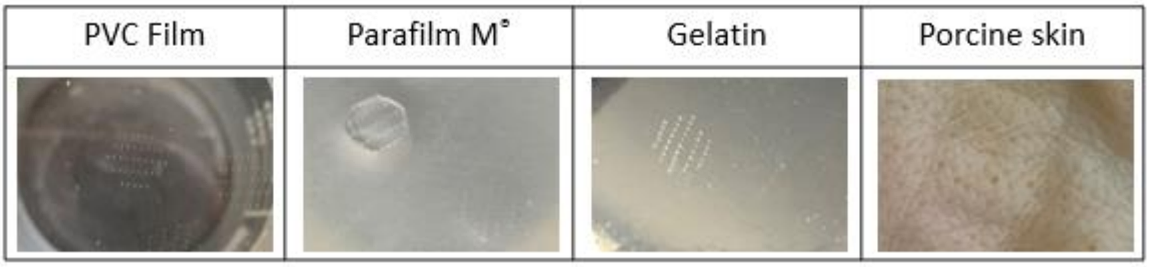

Figure S4: Images of the surfaces (PVC film, Parafilm M®, gelatin, and porcine skin) after compression/puncture test.

Table S2: Statistical *p* values from the measurement analysis of the height and base of the microneedles (comparing formulations with the same extract concentration and the same P407 concentration).

Student *t*-test analysis				*p* value			
Compared formulations		Height	Base	Compared formulations		Height	Base
E1	E2	0.0930	0.0080	G1	G2	0.2600	0.0270
E1	E3	0.4180	0.0012	G1	G3	0.0800	0.3370
E1	E4	0.4830	0.0950	G1	G4	0.0008	0.0023
E1	E7	0.0270	0.5700	G1	G7	0.3000	0.1030
E2	E3	0.0130	0.0730	G2	G3	0.8310	0.0130
E2	E5	0.3640	0.8200	G2	G5	0.9900	0.2100
E2	E8	0.7580	0.1100	G2	G8	0.5200	0.8540
E3	E6	0.0010	0.3200	G3	G6	0.9300	0.0504
E3	E9	0.0050	0.0007	G3	G9	0.2300	0.4700
E4	E5	0.0006	0.4200	G4	G5	0.3600	0.0900
E4	E6	0.0003	0.7200	G4	G6	0.4300	0.3100
E4	E7	0.0010	0.1500	G4	G7	0.6000	0.0290
E5	E6	0.0030	0.5890	G5	G6	0.8500	0.0410
E5	E8	0.2200	0.1400	G5	G8	0.2300	0.1900
E6	E9	0.0030	0.0030	G6	G9	0.3500	0.0900
E7	E8	0.0291	0.2400	G7	G8	0.8900	0.7000
E7	E9	0.0090	0.9970	G7	G9	0.6880	0.0300
E8	E9	0.2230	0.1710	G8	G9	0.9300	0.0100

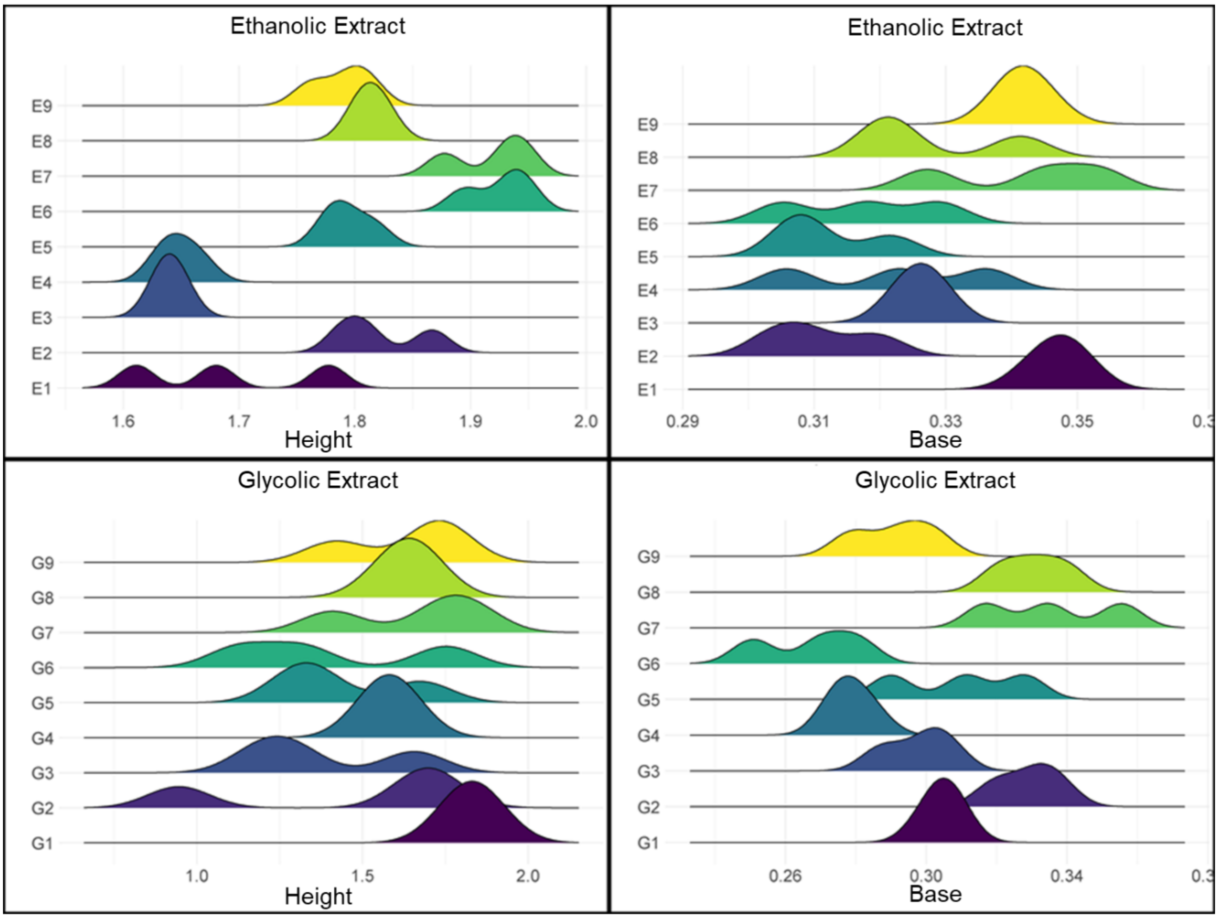

Figure S5: Statistical comparison between means by Student's *t*-test for the analysis of measurements.

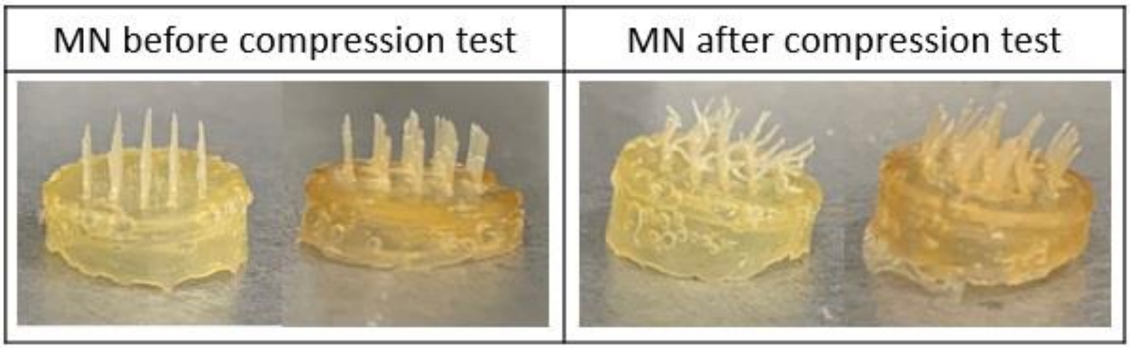

Figure S6: Images of the MN before and after the compression test, focusing on the structure of the tip of the microneedle.
